# Designing efficient photochromic dithienylethene dyads†
†Electronic supplementary information (ESI) available: Comparison between ADC(2) and CAM-B3LYP for a single DTE, conformational study of dyad **1** and basis set effect on the computed optical properties, TD-DFT results for a “normal-inverse” dimer. See DOI: 10.1039/c5sc00856e


**DOI:** 10.1039/c5sc00856e

**Published:** 2015-04-02

**Authors:** Arnaud Fihey, Denis Jacquemin

**Affiliations:** a Chimie Et Interdisciplinarité, Synthèse, Analyse, Modélisation (CEISAM) , UMR CNRS no. 6230 , Université de Nantes , BP 92208, 2, Rue de la Houssinière , 44322 Nantes Cedex 3 , France . Email: denis.jacquemin@univ-nantes.fr; b Institut Universitaire de France , 103, blvd Saint-Michel , F-75005 Paris Cedex 05 , France

## Abstract

Aiming at designing more efficient multiphotochromes, we investigate with the help of *ab initio* tools the impact of the substitution on a series of dimers constituted of two dithienylethene (DTE) moieties, strongly coupled to each other through an ethynyl linker. The electronic structure and the optical properties of a large panel of compounds, substituted on different positions by various types of electroactive groups, have been compared with the aim of designing a dyad in which the three possible isomers (open–open, closed–open, closed–closed) can be reached. We show that appending the reactive carbons atoms of the DTE core with electroactive groups on one of the two photochromes allows cyclisation to be induced on a specific moiety, which leads to the formation of the desired closed–open isomer. Substituting the lateral positions of the thiophene rings provides further control of the topology of the frontier molecular orbitals, so that the electronic transition inducing the second ring closure stands out in the spectrum of the intermediate isomer.

## Abstract 2

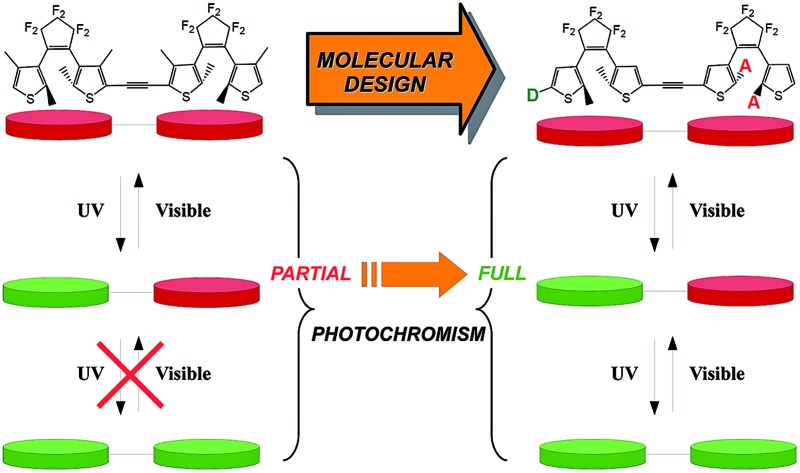
The impact of chemical substitution on the optical properties of *ca.* 30 dithienylethene (DTE) dyads is investigated with first-principles approaches, with the aim to provide useful guidelines for obtaining more efficient DTE multimers.

## 1 Introduction

**Fig. 1:**
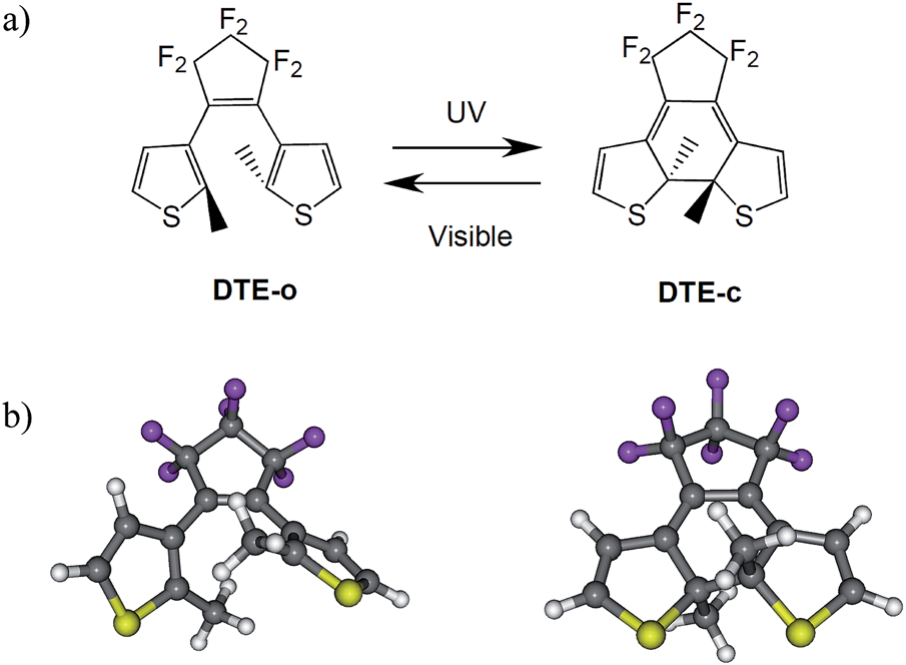
(a) Isomerisation process in a typical DTE. (b) Three-dimension ball and stick representation of **DTE-o** (left) and **DTE-c** (right).

Photochromism in organic compounds brings switching abilities down to the molecular scale.^1^–^3^ Two of the most efficient classes of organic photochromes are azobenzene and dithienylethene (DTE) derivatives, which have been widely studied both experimentally^4^,^5^ and theoretically^6^–^8^ and have been applied in several devices.^9^–^11^ DTEs develop robust switching abilities, thanks to their light-induced thermally irreversible isomerisation between an open form (**o**) that generally absorbs in the UV region only, and a more conjugated closed form (**c**) that absorbs visible light (see Fig. 1). Furthermore, the switching process in DTEs is often doable thousands of times without any loss of efficiency.^12^

The two isomers of a DTE photochrome can be viewed as “off” and “on”, or “0” and “1” states, and used to store optical information, modulate conductance between two contacts,^13^ or induce movement in flexible polymer matrices.^14^ Beyond the binary functionality of the isolated photochromes, systems combining two or more photochromes have been reported during the last decade.^15^–^19^ The multiphotochromic DTE assemblies are by far the most studied but the design of fully-functional objects has encountered several issues.^20^ In systems in which all DTEs are weakly coupled each unit behaves independently, as in an isolated photochrome, and one is stuck with a binary object. For example, in the hexamer reported in ref. 19, the six ring closures are simultaneous and the absorption properties of the hexamer are not different from those of the isolated units, but for a six-fold increase in the intensity of the hallmark visible band. In addition, when the linker separating the different DTEs prevents electronic communication, the DTEs close in an uncontrolled fashion. Consequently the partially isomerised compound(s) cannot be easily isolated and the number of states does not increase with respect to the isolated photochrome. This outcome has been observed in DTE dyads built with a quinonediimine,^21^ a perylenediimide,^22^ a porphyrin core,^23^ as well as in H-bonded structures.^24^ Clearly, if one wishes to obtain a multiphotochromic system that presents emerging properties, it is necessary for the different fragments to be influenced by the form (open or closed) of their neighbor(s). This can be achieved by using a linker that allows significant electronic or steric interactions.^25^–^30^ For instance, in ref. 28 and 29 the phenyl bridge separating the two identical DTEs provides sufficient conjugation and the partially closed isomer can be isolated before the formation of the doubly-closed isomer. However, the closure of all the DTEs in conjugated dyads is not always reached (see, for example, dimers in ref. 25 and 30 possessing respectively an ethynyl and a bisthiophene bridge) due to an excessive amount of electronic “through-bond” interactions. This outcome has been rationalised in previous theoretical studies:^31^ when the DTEs are linked through a short and conjugated bridge, the electronic structure of the open unit in the hybrid closed–open isomer is often perturbed to a point where the photochromic characteristics of the open DTE are lost. The nature of the bridge is thus one of the critical parameters guiding the amount of electronic communication between the photochromes, though steric hindrance has also been highlighted as a limiting factor in specific cases.^32^,^33^

Although several studies have been conducted, on the one hand, to rationalise the optical signature and photochromic behaviour in DTE multimers,^20^,^31^ and, on the other hand, to predict the effects of the chemical substitution on the UV-visible spectrum of isolated DTEs,^34^–^37^ no investigation of the impact of chemical substitution on the photochromism of dyads has appeared yet. This is surprising, as chemical substitution is a promising approach for controlling the optical properties of the switch, *e.g.*, to maximise the contrast between the two isomers.^38^ In this work we propose unprecedented substitution patterns to design more effective DTE dimers. Indeed we undertake the tuning of the electronic properties of dyads by testing a wide range of chemical substitutions. A series of *ca.* 30 compounds have been modelled using time-dependent Density Functional Theory (DFT).^39^,^40^ After rationalising the observed partial photochromism in an available ethynyl-bonded dimer,^25^ we propose a strategy to bypass the pinpointed bottlenecks while conserving this linker that guarantees a strong electronic communication between the units constituting the dimer.

## 2 Methods

A two-step computational scheme has been used in this work. This approach has been shown to yield both accurate ground-state structures and absorption properties in both isolated^36^ and coupled^31^,^32^,^41^ DTE derivatives. The PBE0^42^ global hybrid and the CAM-B3LYP^43^ range-separated hybrid functionals are associated, to optimise the ground state geometry with the 6-311G(d,p) atomic basis set, and to compute the optical properties with the 6-311+G(2d,p) atomic basis set, respectively. The CAM-B3LYP functional that includes a growing amount of exact exchange with inter-electronic distance allows the comparison of various delocalised compounds on a physically well-grounded basis. This choice of the CAM-B3LYP functional is justified in the ESI† by comparing transition energies, oscillator strengths and the nature of the lowest excited-state of open and closed DTEs to wavefunction results obtained at the ADC (Algebraic Diagrammatic Construction) level. For all criteria the agreement is obvious (see ESI†). To screen the large number of DTE dyads under study, the smaller atomic basis set 6-31G(d) was then used for both steps. When comparing theoretical absorption properties to experimental measurements, the solvent is taken into account using the well-known Polarisable Continuum Model (PCM) model.^44^ All theoretical spectra shown in this work are convoluted with a Gaussian function presenting a width at half height of 0.3 eV. All calculations have been performed using the Gaussian09 program.^45^

## 3 Results and discussion

### 3.1 Rationalisation of experimental findings

**Fig. 2:**
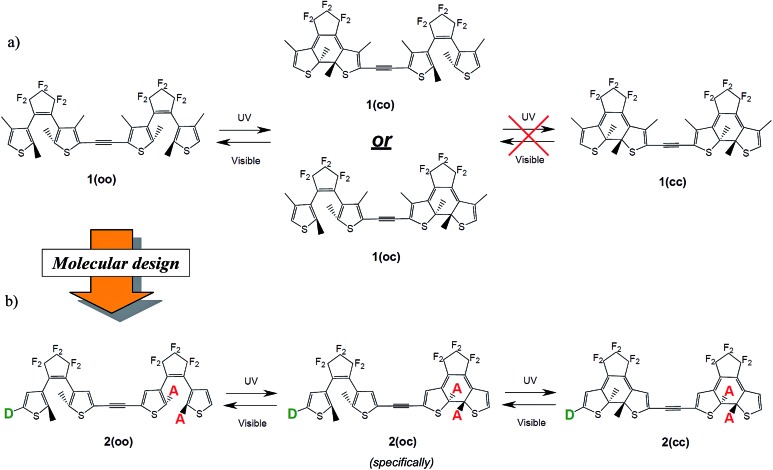
(a) Partial isomerisation process for **1** as observed in ref. 25. (b) Full and controlled isomerisation process as expected in the proposed dyads. **D** and **A** stand, respectively, for donor and acceptor groups.

In symmetric photochromic dimers, one expects the presence of three isomers, namely the fully open, mixed closed–open and fully closed derivatives, noted respectively as **oo**, **co** (or **oc**) and **cc** (see Fig. 2). However in **1** only one of the two DTEs can be closed.^25^ This partial photochromism was originally attributed to a potential quenching of the excited state of the open DTE through an energy transfer towards the closed DTE. Later, theoretical studies shed light on another facet of these types of dyad.^20^,^31^ Indeed, in the mixed **co** isomer, the computation of the electronic transitions and the analysis of the topology of the virtual orbitals involved in the key excited states highlighted that there is no efficient pathway to populate an orbital showing a bonding interaction for the to-be-formed C–C bond. Consequently, such a bond cannot be created photochemically.^46^ This result is a consequence of the use of a short and conjugated linker: in **co** the electronic structure of the open DTE is very strongly influenced by the presence of the closed DTE.

Table 1 compares the experimental and theoretical absorption properties for the three isomers of **1**. For this dimer the structure with DTEs in an *anti* conformation is slightly more stable than that in the *syn* conformation (see the ESI†), and is investigated hereafter. The CAM-B3LYP/6-311+G(2d,p) results are in very good agreement with the experimental absorption spectra, with a deviation of a few nanometers for the transitions in both **1(oo)** and **1(co)** (see Table 1). The use of a smaller atomic basis set, 6-31G(d), induces a systematic hypsochromic shift limited to *ca.* 0.05 eV for the three isomers (see the ESI†). The contribution of the molecular orbitals in the electronic transitions is similar with the two basis sets and the same holds for the absorption spectra. The smaller basis set is therefore suitable for monitoring the effects of the substitutions on the optical properties.

**Table 1:** Theoretical and experimental^25^ optical properties for the different isomers of **1** in hexane. Theoretical values have been obtained at the CAM-B3LYP/6-311+G(2d,p) level. *λ* is the wavelength (in nm) and *f* the oscillator strength of the electronic transition. Δ*E*_exp/theo_ is the difference between theoretical and experimental energies, in eV. H and L stand for the HOMO and LUMO, respectively

Isomer		*λ*	Δ*E*_exp/theo_	*f*	Composition
**1(oo)**	exp	320			
		*ca.* 270			
	theo	328	–0.09	1.00	H → L (63%)
					H → L + 1 (16%)
					H → L + 2 (14%)
		272	+0.03	0.09	H – 2 → L + 1 (18%)
					H – 1 → L + 2 (18%)
					H → L + 3 (13%)
					H → L + 6 (11%)

**1(co)**	exp	584			
		*ca.* 365			
	theo	576	+0.03	0.49	H → L (94%)
		365	0.00	0.49	H – 2 → L (17%)
					H – 1 → L (71%)

**1(cc)**	exp	—	—	—	
	theo	663		0.87	H → L (85%)
					H – 1 → L + 1 (13%)
		377		0.48	H – 2 → L (68%)
					H – 1 → L + 1 (11%)
					H – 3 → L + 1 (14%)

**Fig. 3:**
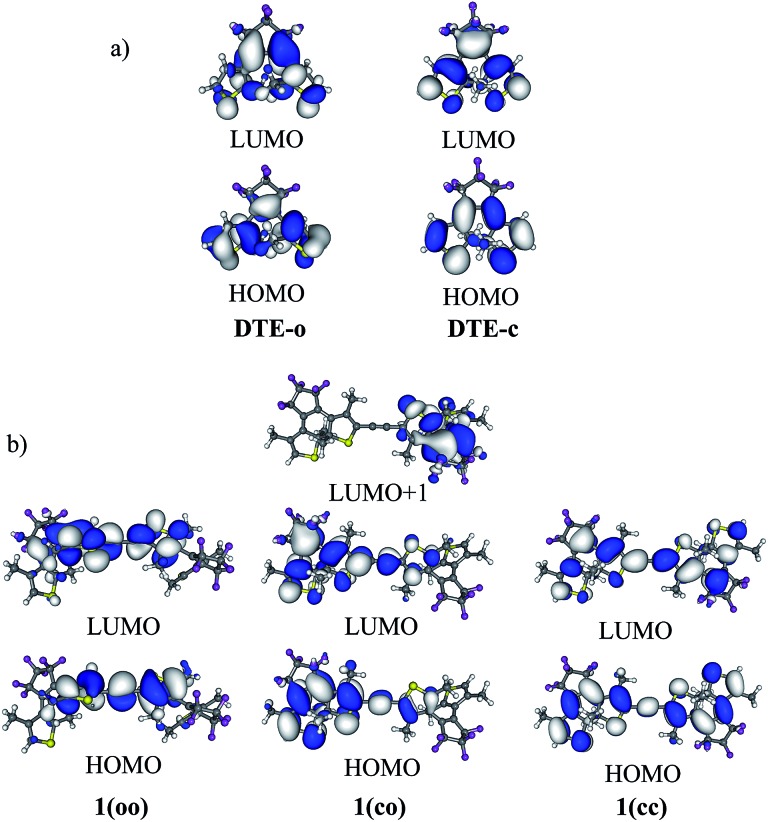
(a) HOMO and LUMO of **DTE-o** and **DTE-c**. (b) HOMO and LUMO of the different isomers of **1** (cut-off = 0.02 a.u.).

In **1(oo)**, the spectrum is dominated by the first and intense transition in the near UV, corresponding mainly to a HOMO → LUMO transition. The LUMO of **1(oo)** is located mainly on the central part of the dyad (see Fig. 3), and a close look at the topology reveals bonding interactions between the reactive carbon atoms for both DTEs. The LUMO can therefore be denoted a “photochromic orbital” and UV irradiation (around 330 nm) induces an excitation to a state suitable to initiate electrocyclisation, which is consistent with the experimental findings. The transition towards a photochromic orbital will be referred to as a “photochromic transition” hereafter. Concerning the **co** isomer, two main transitions contribute to the absorption spectrum: a HOMO → LUMO transition in the visible region, and a combination of a HOMO – 1 → LUMO (71%) and a HOMO – 2 → LUMO (17%) transition in the near UV domain (365 nm). As stated above, the LUMO is centred on the closed DTE, and the first virtual orbital centred on the open moiety and possessing a photochromic topology (that is similar to the LUMO of the isolated open DTE with a bonding interaction between the reactive carbon atoms) is the LUMO + 1 (Fig. 3). However, the LUMO + 1 is not involved significantly in the computed electronic transitions above 300 nm. The composition of the computed electronic transitions and the topology of the molecular orbitals of the **co** isomer are thus consistent with the loss of photochromism and the impossibility of forming the **cc** isomer experimentally.

### 3.2 Substitution strategy

**Fig. 4:**
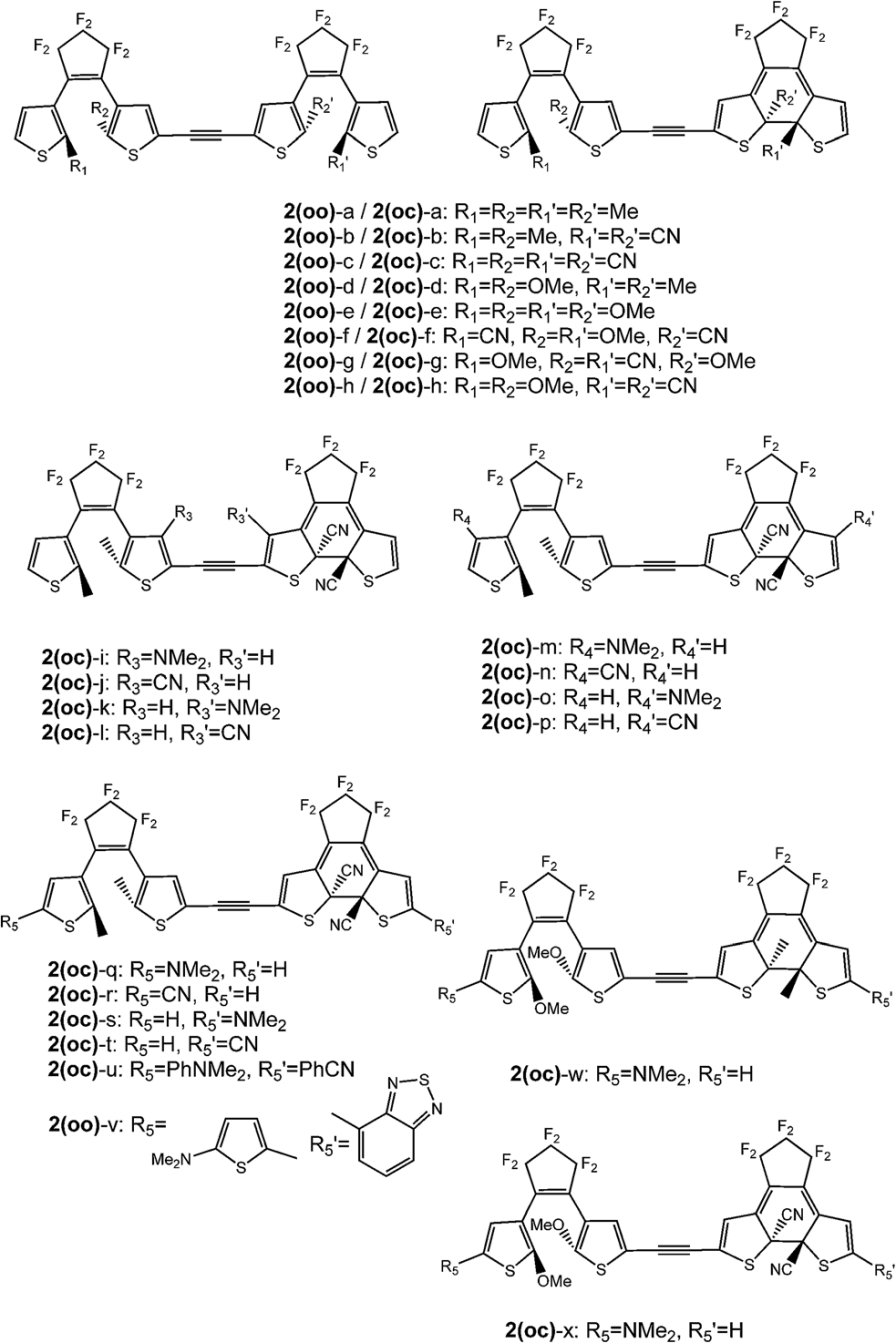
DTE dyads under investigation.

Photochrome **1** is a typical illustration of the pitfalls encountered when linking two DTE moieties through a highly conjugated and short linker. The loss of the photochromic transition in the **co** isomer should clearly be overcome to design dimers conserving the photochromic features of their components and allowing a significant communication between the subunits so that **co** and **cc** optical responses differ significantly. To elucidate the role of the linker on the photoactivity, and to determine if the second ring closure is systematically beyond reach with an ethynyl linker, we studied a series of possible modifications of the DTE moieties. We established a sequential three-step substitution strategy, based on three criteria allowing an improved control of the multiphotochrome:

(1) To obtain three isomers, **oo**, **co** and **cc**, one first wishes to reach stepwise photochromism, that is to guarantee that the two DTEs do not isomerise simultaneously. To attain this goal a dissymmetric substitution can be applied to induce the cyclisation of a specific DTE. For instance, adding acceptor groups on the reactive carbons on one DTE (see Fig. 2) localises the photochromic HOMO → LUMO transition on one given photochrome. By substituting in these R_1_ and R_2_ positions (see Fig. 4), one also takes advantage of the different natures of the reactive carbon atoms in the open (sp^2^, in the π conjugation pathway) and in the closed (sp^3^, out of the π conjugation pathway) isomers to modulate the impact of the substituent in the open and closed isomers. With such a strategy, one can induce a first closure on an open DTE, whilst not impacting significantly the electronic structure of the obtained **co** isomer.

(2) Full ring closure: after the initial electrocyclisation, the LUMO is “trapped” on the most conjugated fragment that is on the closed DTE and the linker. The first virtual orbital that presents a photochromic shape centred on the remaining open DTE can only be found at higher energy (at least, LUMO + 1). Tuning the topologies and energies of the orbitals with substituents is necessary for obtaining a photochromic virtual orbital localised on the open DTE in the **co** isomer, and accessible by irradiation at *ca.* 300 nm (310–320 nm irradiation wavelengths are typically used experimentally). In this regard, it can be intuited that the addition of electroactive groups on the remaining open DTE (see Fig. 2) could decouple the photochromic virtual orbital centred on this unit from the HOMO and LUMO localised on the closed moiety. In this way a photochromic transition could stand out more clearly in the **co** spectrum.

(3) To ensure a maximal optical sensitivity and contrast, we also assessed the spectral differences between the three isomers in the investigated series, by comparing their respective *λ*_max_ (maximum absorption wavelength) values. This criterion implies in particular that the shift between the **co** and **cc** visible absorption bands is non-negligible.

Those three criteria are consecutively investigated in the next sections, considering **2** as a raw skeleton (see Fig. 4). We focus on the electronic effects brought by the substituents, using both electron donor (NMe_2_ or OMe) and acceptor (CN) groups. Additionally, both larger conjugated donor (Ph–NMe_2_, thiophene–NMe_2_) and acceptor groups (Ph–CN, benzothiadiazole) have been tested. The substitution strategy follows the design illustrated in Fig. 2 and the results are presented as follows: the impact of the substitution on the reactive carbon atoms is first studied solely on **oo** isomers, then on **oc** isomers, and the effects of the side groups added to the thiophene rings of the latter are finally detailed. In parallel, compound **3** combining one “normal” and one “inverse” DTE was tested and the results are given in the ESI.†

The key features in the electronic structure of this series of dyads is the presence, or not, of a first low-lying virtual orbital presenting a photochromic topology, and its involvement, or not, in the absorption band irradiated experimentally. We thus focus below on the photochromic transitions.

### 3.3 Controlling the initial cyclisation in fully open isomers

First we considered the substitution of the reactive carbon atoms (R_1_, R_2_, R_1_′ and R_2_′ in Fig. 4). Experimental works with substitution on these positions have been published for DTEs, and it was shown that adding methoxy^47^,^48^ or butoxy^49^ groups induces a strong decrease of the ring-opening photochemical (cycloreversion, **c** → **o**) quantum yield, whereas adding acceptor groups (CN) strongly increases this yield.^50^ In contrast, these substitution patterns have negligible effects on the photoinduced cyclization of the DTEs.^47^,^48^,^50^

**Fig. 5:**
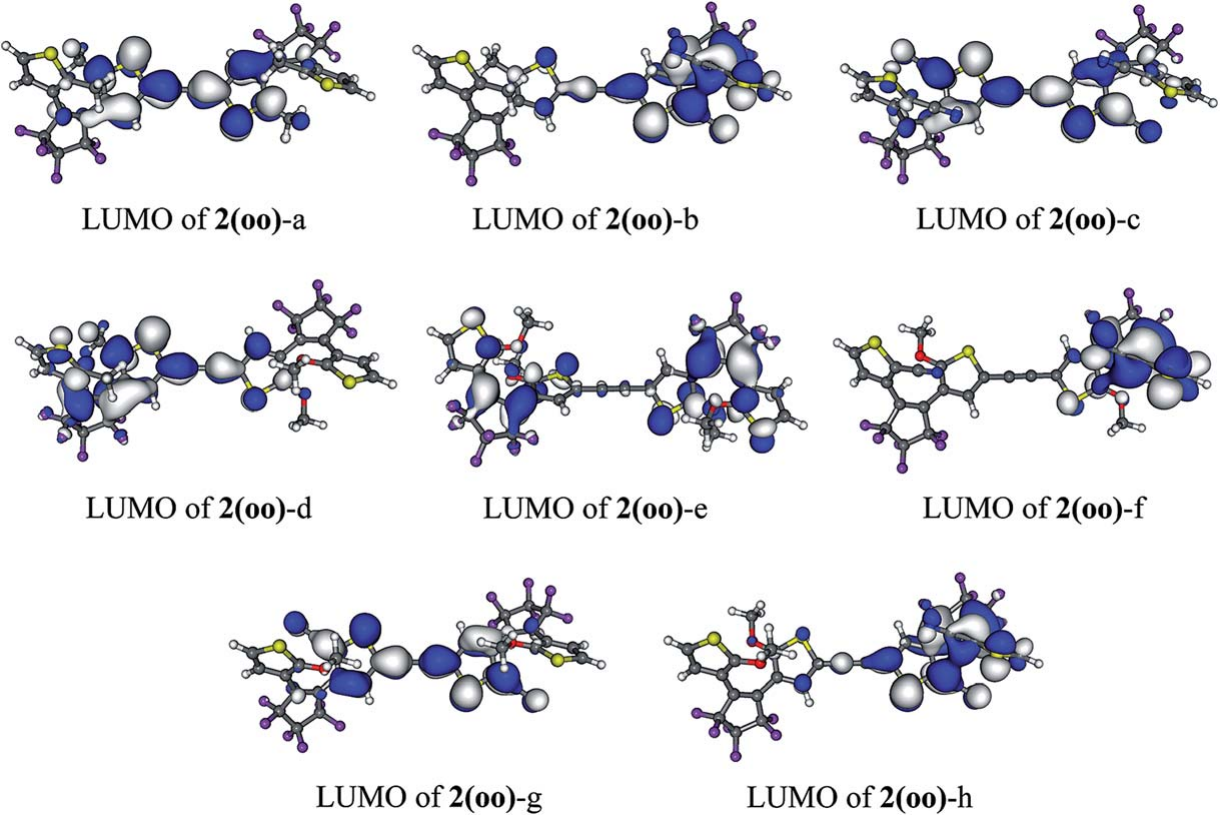
First virtual photochromic orbital in **2(oo)** derivatives (cut-off = 0.02 a.u.).

The absorption properties of the **oo** compounds under investigation are similar to those of the **1(oo)** dyad. The first excitation corresponds to a HOMO → LUMO transition and thus the localisation of the LUMO the key for selective ring closure. Table 2 lists the photochromic transition in the different **2(oo)** derivatives, obtained by replacing the methyl group on the reactive carbons by OMe or CN groups. The substitutions have been considered on one or both DTEs, as substituting only one side of the dimer may induce an interesting dissymmetrisation of the electronic structure. When a symmetric substitution pattern is applied, the first virtual orbital is delocalised upon the central linker and one of the thiophene rings of each photochrome, similarly to the LUMO of **1(oo)**. This is indeed the outcome when all substituents are Me [**2(oo)**-a, see Fig. 5], CN [**2(oo)**-c] or OMe [**2(oo)**-e]. The LUMO presents a photochromic character and the HOMO → LUMO photochromic transition may indifferently induce the cyclisation of the DTEs with *a priori* the same efficiency. By contrast, differentiating R_1_ and R_2_ from R_1_′ and R_2_′ induces a site-specific localisation of the first low-lying virtual orbitals. For instance, in **2(oo)**-b with R_1_ = R_2_ = Me and R_1_′ = R_2_′ = CN, the LUMO is centred on the CN-substituted DTE (see Fig. 5). In **2(oo)**-d, adding donor groups to one DTE also grants selectivity: the two first virtual orbitals are purely centred on the Me-substituted DTE and the destabilised LUMO + 2 is found on the DTE bearing the methoxy moieties. When donor groups are attached to one DTE and acceptor groups to the other [**2(oo)**-h], the same result is reached: the stabilised LUMO is logically localised on the open switch bearing CN groups. The LUMO + 1 is again localised on this part of the dimer and the LUMO + 2 is the first photochromic virtual orbital appearing in the OMe-substituted DTE. For these three dissymmetric dimers [**2(oo)**-b, **2(oo)**-d and **2(oo)**-h], the first photochromic orbital is clearly localised on a single DTE and one can therefore selectively close this DTE through an appropriate irradiation. The LUMO + 2, centred on the other DTE, is involved in higher-lying (below 300 nm) S_0_ → S_3_ or S_0_ → S_4_ transitions that present much lower intensities. It is therefore clear that irradiating in the S_1_ region should induce a fully selective switching.

**Table 2:** Effect of the substitution on the reactive carbon atoms on the computed S_1_ excited state for different derivatives of **2(oo)**. In addition to the wavelength (*λ*/nm) and oscillator strength (*f*), the localisation of the LUMO photochromic orbital is also given, together with the orbital composition of this transition

	DTE with LUMO	*λ*	*f*	Composition of S_1_
**2(oo)**-a	Both	321	1.00	H → L (81%)
**2(oo)**-b	CN-substituted	350	0.83	H → L (79%)
**2(oo)**-c	Both	350	0.99	H → L (89%)
**2(oo)**-d	Me-substituted	330	1.00	H → L (58%)
				H → L + 1 (31%)
**2(oo)**-e	Both	302	0.77	H – 1 → L (30%)
				H → L + 1 (41%)
**2(oo)**-f	First DTE	363	0.16	H → L (78%)
	*L + 1: second DTE*	*355*	*0.13*	*H → L + 1 (78%)*
**2(oo)**-g	Both	351	0.28	H → L (67%)
**2(oo)**-h	CN-substituted	368	0.90	H → L (72%)

In both **2(oo)**-f and **2(oo)**-g the substitution pattern is symmetric but one donor and one acceptor is added to each DTE. In **2(oo)**-f the LUMO and LUMO + 1 are not centred on the same DTE and the transition to the two corresponding states, S_1_ and S_2_, are very close in energy and possess a similar intensity: irradiating in this region would indifferently close one of the two DTEs. In **2(oo)**-g, the S_1_ photochromic transition populates a virtual orbital delocalised on both DTEs and the photocyclisation is also not selective.

In summary, introducing either acceptor or donor groups on the reactive carbons of one DTE induces a clear dissymmetrisation of the electronic structure of the **oo** isomer, the LUMO becoming localised on one side of the dyad. Compound **2(oo)**-b is a straightforward example: the CN groups placed on the reactive carbon atoms yield a stabilised LUMO localised on the substituted DTE. The lowest lying and most intense electronic transition can therefore be used to selectively close this DTE, which guarantees the formation of a specific **oc** isomer.

### 3.4 Mixed open/closed dimer: how to close the second photochrome?

Let us now consider the bottleneck of the second cyclisation process. We are searching for an electronic transition that significantly populates a virtual orbital possessing a photochromic shape on the open DTE. As quantitative criteria, we have selected wavelengths larger than 300 nm, oscillator strengths exceeding 0.10 and a ratio of population of the photochromic orbital of at least 25%. These thresholds were chosen on the basis of previous theory/experiment comparisons,^20^ so that the selected photochromic transitions unambiguously stand out in the computed spectrum. For the series of **oc** dimers under investigation, the photochromic transitions found with TD-DFT are detailed in Table 3.

**Table 3:** Photochromic electronic transitions in the different **oc** isomers: the nature of the photochromic orbital, numbering of the state, absorption wavelength (*λ*/nm), oscillator strength (*f*) and the ratio of population of the photochromic orbital within this transition are given. PO stands for photochromic orbital. Transitions considered as efficient (see text) are in bold

	PO	State	*λ*	*f*	Population ratio (%)
**2(oc)**-a	L + 1	S_4_	302	0.08	16
		S_5_	287	0.16	57
**2(oc)**-b	L + 1/L + 2	S_3_	320	0.29	14
		S_6_	282	0.12	84
**2(oc)**-c	L + 1	**S_3_**	**331**	**0.16**	**46**
		**S_5_**	**303**	**0.20**	**36**
		**S_6_**	**299**	**0.11**	**21**
		S_10_	268	0.19	48
**2(oc)**-d	L + 1	S_4_	302	0.06	22
		**S_5_**	**300**	**0.21**	**66**
**2(oc)**-e	L + 1	S_5_	299	0.20	6
**2(oc)**-f	L + 1	S_2_	346	0.55	17
		S_3_	340	0.02	74
**2(oc)**-g	L + 1	**S_4_**	**331**	**0.47**	**41**
		S_5_	314	0.20	20
**2(oc)**-h	L + 2	S_7_	295	0.13	72

**2(oc)**-i	L + 1	S_6_	303	0.14	15
		S_10_	279	0.23	44
**2(oc)**-j	L + 2	—	—	—	—
**2(oc)**-k	L + 1/L + 2	**S_3_**	**327**	**0.10**	**44**
		S_7_	288	0.10	65
**2(oc)**-l	L + 2	S_8_	278	0.10	83

**2(oc)**-m	L + 1/L + 2	S_3_	320	0.28	18
**2(oc)**-n	L + 1/L + 2	**S_3_**	**319**	**0.36**	**26**
**2(oc)**-o	L + 1/L + 2	S_7_	282	0.12	56
**2(oc)**-p	L + 2	S_8_	279	0.12	65

**2(oc)**-q	L + 1/L + 2	S_5_	319	0.32	13
**2(oc)**-r	L + 1	S_6_	285	0.07	68
**2(oc)**-s	L + 1	S_3_	326	0.56	10
**2(oc)**-t	L + 2	S_9_	279	0.12	79
**2(oc)**-u	L + 2	S_6_	318	0.05	78
**2(oc)**-v	L + 2/L + 3	**S_5_**	**336**	**0.39**	**63**
**2(oc)**-w	L + 1	S_4_	303	0.09	72
		S_5_	301	0.20	18
**2(oc)**-x	L + 2	**S_6_**	**301**	**0.18**	**53**
		S_7_	299	0.06	39

In all the **oc** compounds, the LUMO is, as expected, localised on the most conjugated closed side or on both the closed DTE and the linker. Only orbitals higher in energy can be centred on the open moiety and be potentially useful for inducing cyclisation of the open switch. For all investigated **2(oc)** derivatives, the photochromic orbital with a bonding interaction between the reactive carbon atoms is either the LUMO + 1 or the LUMO + 2 orbital. However, in most **2(oc)** derivatives, these photochromic orbitals are weakly involved in the computed electronic transitions, or when they are, the corresponding oscillator strength is rather weak. This outcome is probably sufficient to discard a possible second photocyclisation. In fact, the presence of an efficient transition fulfilling the above cited criteria is more an exception than a rule.

#### 3.4.1 Substitution on the reactive carbon atoms

First, derivatives of **2(co)** substituted at the reactive carbon atoms by Me, CN or OMe groups have been considered. When the dimer is not symmetric, we considered the hybrid closed/open isomer resulting from the initial selective electrocyclisation (see Section 3.3). The photochromic transitions in this series of compounds [**2(oc)**-a to **2(oc)**-h] are listed in Table 3. Even for symmetric substitution, the presence of a closed and an open photochrome induces an asymmetric electronic structure with molecular orbitals that can be localised either on the highly conjugated part (closed DTE and linker) or on the weakly conjugated part (open DTE). This can be seen from the molecular orbitals of **1(co)** in Fig. 3.

**2(oc)**-a does not possess any photochromic transition fulfilling the selected criteria. The same holds for both the dissymmetric CN-substituted **2(oc)**-b and the donor–acceptor **2(oc)**-h. Both the LUMO + 1 and LUMO + 2 of **2(oc)**-b are photochromic orbitals but they are not significantly involved in the calculated electronic transitions of interest (*ca.* 15% weight). The photochromic LUMO + 2 of **2(oc)**-h is not present in transitions above 295 nm.

Both symmetric dyads **2(oc)**-c and **2(oc)**-g present intense photochromic transitions peaking at *ca.* 330 nm. The photochromic LUMO + 1 is populated up to 46% and 41% for **2(oc)**-c and **2(oc)**-g, respectively. These compounds present an efficient pathway to a photochromic excited state useful to induce the **co** → **cc** step, though as all-symmetric dyads they do not grant selectivity in the first **oo** → **co** step. Substitution of the reactive carbon atoms of the dimer is consequently an efficient strategy to control not only the first switching, but also, in some specific cases, to promote the second one. Such a dyad can be achieved by a symmetric synthesis which is clearly appealing.

**Fig. 6:**
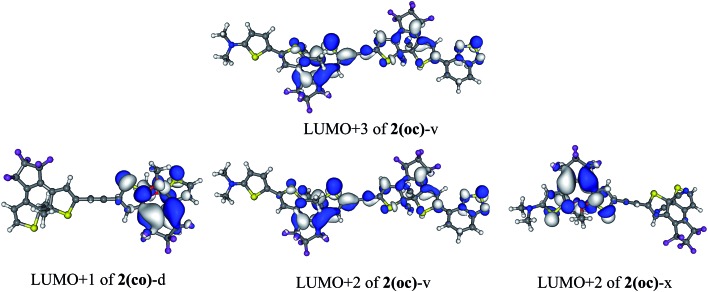
First virtual photochromic orbital of **2(co)**-d, **2(oc)**-v and **2(oc)**-x (cut-off = 0.02 a.u.).

To selectively form the targeted **oc**, the dissymmetric **2(oc)**-d is a useful option. It can be selectively closed in the first step and presents a photochromic LUMO + 1 centred on the OMe-substituted DTE for the second closure (see Fig. 6). This LUMO + 1 is like the LUMO of an isolated open DTE, and the intense 300 nm transition includes a blend of HOMO → LUMO + 1 (22%) and HOMO – 1 → LUMO + 1 (44%) components. On the basis of this orbital analysis, irradiation at *ca.* 300 nm could trigger the formation of the **2(cc)**-d isomer. From the above results, we conclude that favouring an energetic decoupling between the LUMO and LUMO + 1 orbitals helps maintaining a photochromic transition in the **oc** isomers.

#### 3.4.2 Substitution on the thiophene rings

In this second series, the thiophene rings are substituted by a NMe_2_ donor or a CN acceptor group. Three positions are considered, either on the lateral α (R_5_) or β (R_4_) positions of the ring, or on the “internal” position (R_3_, see Fig. 4). We consider only **oc** dimers granting the control of the site of the first cyclisation (see previous section). Therefore **2(oc)**-b possessing CN(Me) groups on the closed (open) DTE was primarily used as a basis, though **2(oc)**-d and **2(oc)**-h dyads were also considered.

Table 3 lists the photochromic transitions of the **2(oc)** derivatives with different substitutions at the R_3_/R_3_′ positions [compounds **2(oc)**-i to **2(oc)**-l]. By comparing the results for these compounds to the optical properties of the parent **2(oc)**-b dimer, it is clear that the substitution on R_3_ and R_3_′ does not significantly impact the topology of the frontier orbitals, nor changes the relative energy levels. None of the substitution patterns allows an intense electronic transition to be obtained below 300 nm with a large contribution of a photochromic orbital. The most valuable compound in this series is **2(oc)**-k presenting an NMe_2_ group on the thiophene of the closed DTE: the S_3_ state, reachable by irradiation at 327 nm, populates the LUMO + 1 and LUMO + 2 photochromic orbitals with 28% and 16% weights, respectively, but the associated electronic transition is rather weak (*f* = 0.10).

Let us now consider substitutions at R_4_/R_4_′ [**2(oc)**-m to **2(co)**-p] and of R_5_/R_5_′ [**2(co)**-q to **2(co)**-x] (see Table 3). In the first series no drastic enhancement of the presence of the photochromic transition is noted: substitution in the β position of the thiophene rings has little impact. Nevertheless, **2(oc)**-m and **2(oc)**-n, that respectively possess an NMe_2_ and a CN group on the open DTE, exhibit photochromic transitions at *ca.* 320 nm. These transitions involve photochromic orbitals up to 18% for **2(oc)**-m and 26% for **2(oc)**-n, that is more than in the parent **2(oc)**-b. The fact that both donor and acceptor substituents lead apparently to the same behaviour was tentatively ascribed to an increase of the conjugation length, by comparison with model derivatives (not shown).

Photochromic transitions of the dimers substituted at the α position of the thiophene, **2(co)**-q to **2(co)**-x, are also reported in Table 3. A first sub-series, **2(oc)**-q to **2(oc)**-u, derives from **2(oc)**-b. For those dimers the most efficient structures are **2(oc)**-q and **2(oc)**-s, corresponding to the addition of a donor on the thiophene of the open and closed sides, respectively. Both dyads exhibit a photochromic transition around 320 nm that is very intense [*f* = 0.32 for **2(oc)**-q and 0.56 for **2(oc)**-s], though the populations of the photochromic orbital are only 13% and 10%, respectively. They can thus hardly be considered as fully suited to ensure the second photocyclization. Dimer **2(oc)**-u possesses larger substituents (Ph–NMe_2_ and Ph–CN respectively on the R_5_ and R_5_′ positions) but this strategy does not yield improved properties.

Two additional compounds, **2(oc)**-w and **2(oc)**-x, have been studied. In these dyads the addition of NMe_2_ at R_5_ is combined with another pattern for the R_1_, R_2_/R_1_′, R_2_′ positions, *i.e.* two OMe on the open DTE and two Me on the closed one [**2(oc)**-w], or two OMe for the open DTE and two CN for the closed one [**2(oc)**-x]. Compound **2(oc)**-w is analogous to the already studied **2(oc)**-d, that exhibits an intense photochromic transition (see above). This S_0_ → S_5_ transition is found at the same energy and possesses the same intensity in **2(oc)**-w, but the addition of the lateral donor group on the open moiety leads to a decrease of the weight of the photochromic orbital: 18% in **2(oc)**-w instead of 66% in **2(oc)**-d. In contrast, the twice less intense S_4_ is now mostly (72%) ascribed to the LUMO + 1. Compound **2(oc)**-x is one of the dimers for which the photochromic transition stands out the most in Table 3, and this dyad can therefore be viewed as an interesting candidate for synthesis.

Finally, compound **2(oc)**-v (see Fig. 4), which presents a strong conjugated acceptor on the closed moiety and a strong conjugated donor on the open moiety, was proposed in order to maximize the decoupling between the stabilised LUMO (localised on the closed side) and the destabilised photochromic LUMO + *n* centered on the open side. With this strategy an intense S_0_ → S_5_ photochromic transition at 335 nm (*f* = 0.39) is obtained with major contributions of the photochromic LUMO + 2 and LUMO + 3 (63% in total). Dyad **2(oc)**-v is consequently another good candidate for synthesis.

More generally, for the **oc** dimers, the impact of the substitution on the different positions of the DTEs is beyond a simplistic rationalisation. It is clear that dyads of coupled photoactive molecules present a complex electronic structure making the *a priori* prediction of the evolution of their electronic transitions a difficult task. One should account for the fine interplay between three criteria that make a photochromic transition potentially efficient: energy, intensity and orbital composition. These three characteristics are all modified upon chemical substitution. One clear conclusion nevertheless emerges: the addition of an NMe_2_ group on the open DTE often permits decoupling of the frontier orbitals of the open DTE so that the photochromic orbital is more efficiently populated by irradiation in the 300–330 nm region. This the case for dyads **2(oc)**-d, **2(oc)**-v, and **2(oc)**-x that present the most clear-cut photochromic states. By contrast, adding an acceptor group on the different positions of the thiophenes in the **oc** isomer is counterproductive. As a final remark, one could of course argue that populating these photochromic states could be insufficient as the rapid fall to lower-lying states may take place and potentially deactivate the photoinduced process. We first note that in DTEs the photochromic reactions are extremely fast,^51^,^52^ so that cyclization might remain competitive compared to relaxation. Secondly, such a relaxation process is only efficient when the two states are spatially and energetically close. For **2(oc)**-d, **2(oc)**-v, and **2(oc)**-x, the photochromic state of interest is the first one involving the open DTE, and the deactivation mechanism appears less likely.

### 3.5 Fully closed isomers: optical differentiation

**Fig. 7:**
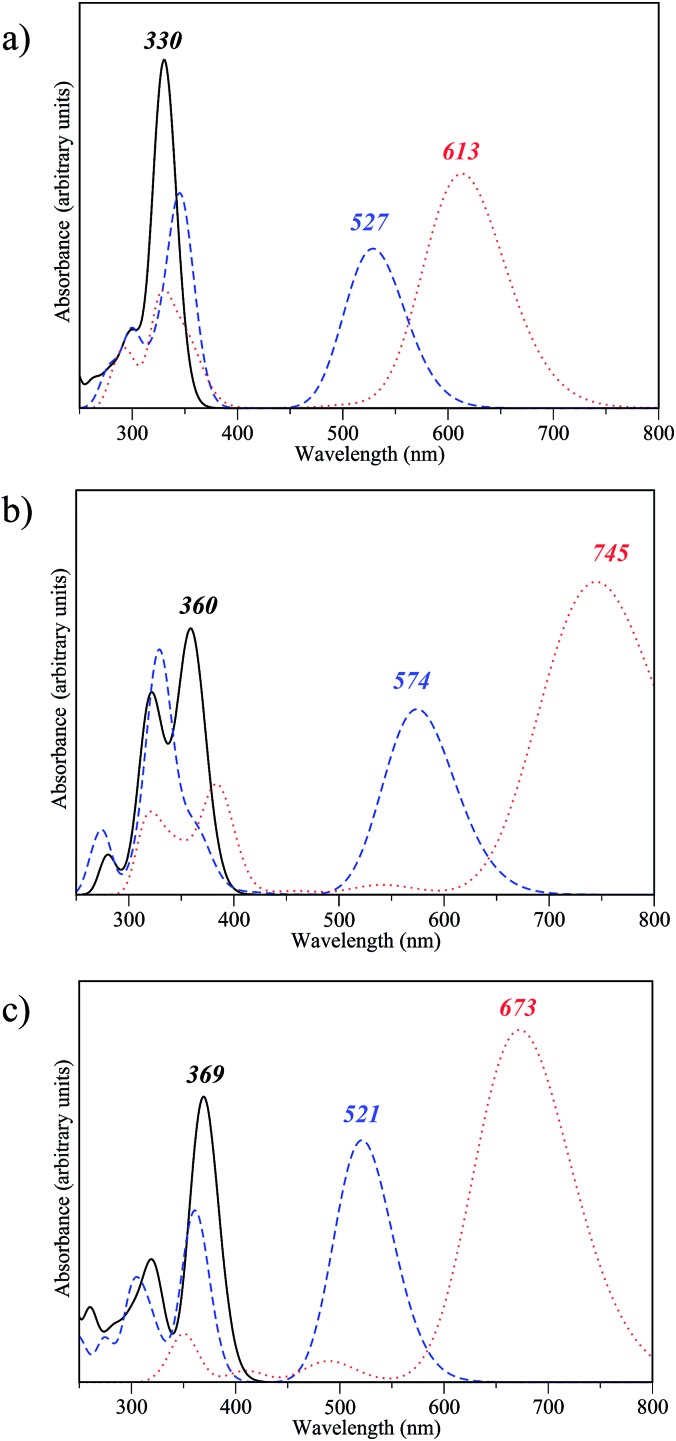
Theoretical spectra for **oo** (solid line), **co** (dashed line) and **cc** isomers (dotted line) of compounds (a) **2**-d, (b) **2**-v and (c) **2**-x.

We now investigate the optical properties of the fully closed form of the DTE dimers pinpointed as efficient during our first two screening steps. Indeed, to be able to easily read the information (the state of the dimer), the three isomers should present distinguishable absorption spectra. Fig. 7 presents the convoluted theoretical absorption spectra of the **oo**, **oc** and **cc** isomers of compounds **2**-d, **2**-v and **2**-x. As expected, in all cases the difference between the *λ*_max_ of the **oo** and **oc** isomers is large as the first electrocyclisation brings a highly conjugated pattern that was absent in the fully open form. In contrast, the differences between **oc** and **cc** are logically smaller, as a saturation of the redshift with the number of conjugated π-electrons takes place. The differentiation of the *λ*_max_ values of **oc** and **cc** is smaller in **2**-d than in **2**-v and **2**-x. The non-negligible difference between the **co** and **cc** absorptions for the latter two is likely related to the presence of π-conjugated lateral substituents, enhancing the conjugation in **cc**. On the basis of our TD-DFT result, those three dimers **2**-d, **2**-v and **2**-x should present experimental optical properties different from the previously synthesised dimer **1**. The three states, **oo**, **oc** and **cc** are accessible, and the spectral contrast is relatively important in both **2**-v and **2**-x.

## 4 Conclusions

The impact of the chemical substitution on the optical properties of *ca.* 30 DTE dyads was investigated with first-principles approaches. These dyads are based on a synthesised system possessing an ethynyl bridge known to exhibit partial closure.^25^ These substitutions follow a molecular design aiming to: (i) selectively close one of the two DTEs in a first step, (ii) bypass the loss of the full photocyclisation occurring in most DTE dyads; and (iii) obtain a sufficient optical contrast between the three isomers. In particular, substituting the reactive carbons of the DTEs, a rarely evoked possibility of substitution in DTE multimers, was found to be an efficient strategy, not only to ensure the selective electrocyclisation of a specific DTE in the dyad but also to increase the predominance in intense transitions of the photochromic orbitals allowing the fully-closed structure to be reached. In the course of this work, several structures stood out as rather simple yet promising substituted dyads. For example adding CN acceptors on all four reactive carbon atoms [**2(oc)**-c] should help reaching the fully closed isomer for a minimal synthetic effort. Of course, dissymmetric patterns can be chosen if one is looking for a selective switching of the first DTE, for instance **2(oc)**-d. Further substitution on the thiophene rings of the two DTEs can bring additional electronic effects helping to reach full closing and the addition of a donor group at the α position of the thiophene ring of the open DTE, in **2(co)**-w and **2(co)**-x, led to an efficient photochromic transition in the mixed isomer. Of course, using orbital and energetic criteria is only a first screening approach, and one needs to investigate other quenching possibilities, as well as the photodynamics to obtain a definitive answer. Clearly, additional studies are to be conducted to assess (i) energy transfer between the two photochromes, as has been done for assemblies of chromophores;^53^ (ii) non-photochromic byproduct formation;^54^ and (iii) excited-state dynamics considering several interacting states. Nevertheless, this work is the first to explore new routes to circumvent the partial photochromism in highly coupled DTE dyads, and our hope is that it provides incomplete yet useful guidelines for future experimental works in the field of DTE multimers.

## Supplementary Material

Supplementary information
