# Supplementary material for: Designing efficient photochromic dithienylethene dyads
Source: Chem Sci. 2015 Apr 2;6(6):3495–504. doi: 10.1039/c5sc00856e (PMC5659146; doi:10.1039/c5sc00856e)
Supplement: Supplementary file 1 [file SC-006-C5SC00856E-s001.pdf]

Electronic Supplementary Material (ESI) for Chemical Science.
This journal is © The Royal Society of Chemistry 2015

Designing efficient photochromic dithienylethene dyads:

Electronic Supplementary Information

Arnaud FIHEY and Denis JACQUEMIN*

Laboratoire CEISAM, UMR CNRS 6230, Université de Nantes, 2 Rue de la Houssinière, BP 92208, 44322 Nantes Cedex 3, France.

E-mail: Denis.Jacquemin@univ-nantes.fr

*To whom correspondence should be addressed

Comparison between CAM-B3LYP and ADC(2) results

Although CAM-B3LYP obviously reproduces the main experimental features we have checked its consistency by comparing the CAM-B3LYP and ADC(2) results obtained for a model DTE, considering both open and closed isomers. Our results are listed in Table S1 and have been obtained with Gaussian09[1] for the TD-DFT part and with Turbomole[2] for the ADC(2) calculations. The relevant MO are displayed un Figure S1. As can be seen the agreement between the two approaches is excellent for all properties. The only unexpected outcome is that the MO composition with ADC(2) seems different from the one obtained with CAM-B3LYP for the open isomer. However, the LUMO of CAM-B3LYP is alike the LUMO+6 of Hatree-Fock (MOs used in the ADC(2) calculation). We note that the Hatree-Fock LUMO to LUMO+5 are spurious Mos, which is typical of HF calculations with large basis sets. Indeed, with 6-31G(d), the ADC(2) LUMO presents the expected canonical shape.

Table S1: Comparison between CAM-B3LYP and ADC(2) spectral properties for the lowest lying excited-state of the open and closed DTE. ΔE is the transition energy given in eV and f is the oscillator strength (au). All results have been obtained with the *aug*-cc-pVDZ atomic basis set.

	Open DTE		Closed DTE	
Property	CAM-B3LYP	ADC(2)	CAM-B3LYP	ADC(2)
ΔE	4.39	4.41	2.56	2.40
f	0.16	0.16	0.12	0.11
Main MO Composition	H-L	H-L+6	H-L	H-L

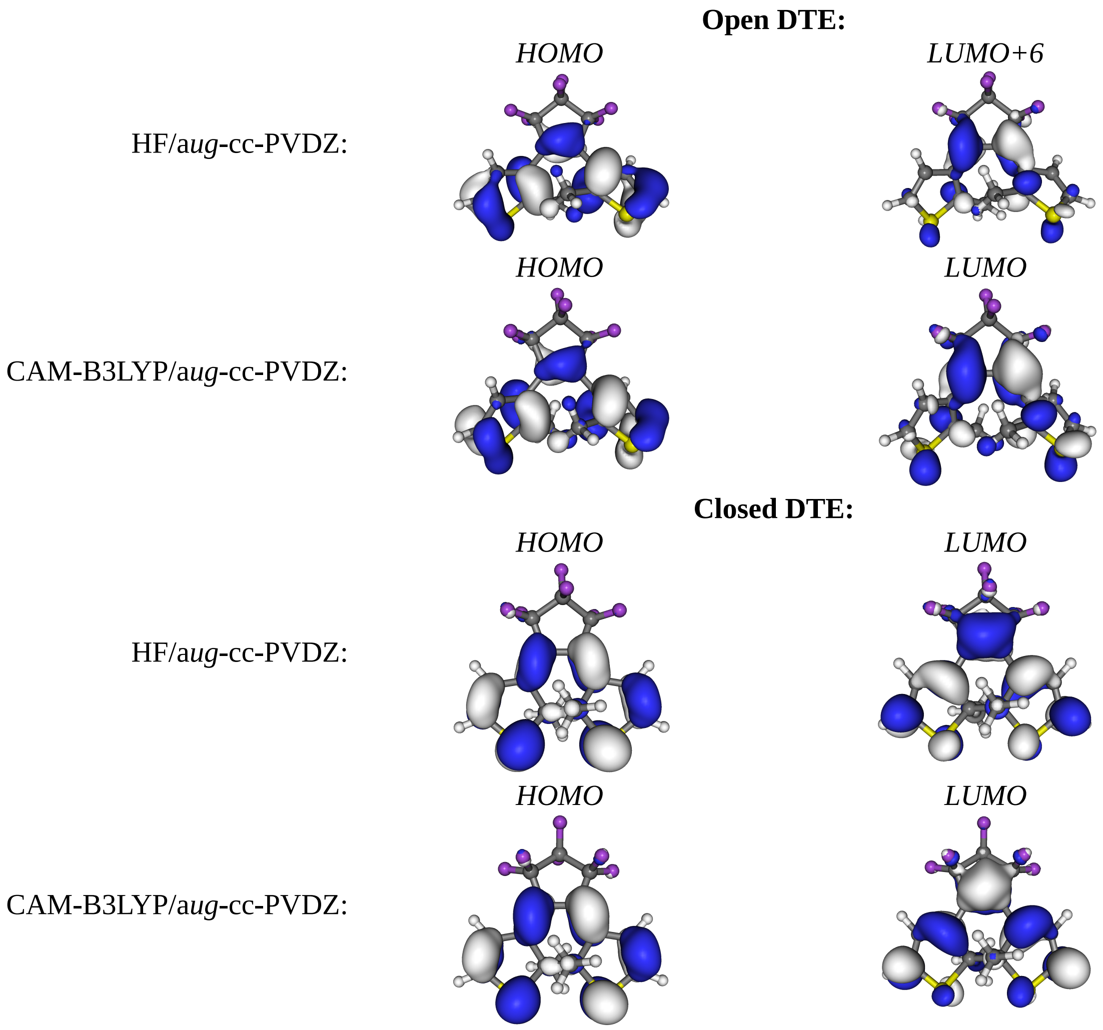

Figure S1: Key molecular orbitals obtained at CAM-B3LYP and HF/ADC(2) levels for open (top) and closed (bottom) model DTEs.

Conformational study of dyad 1

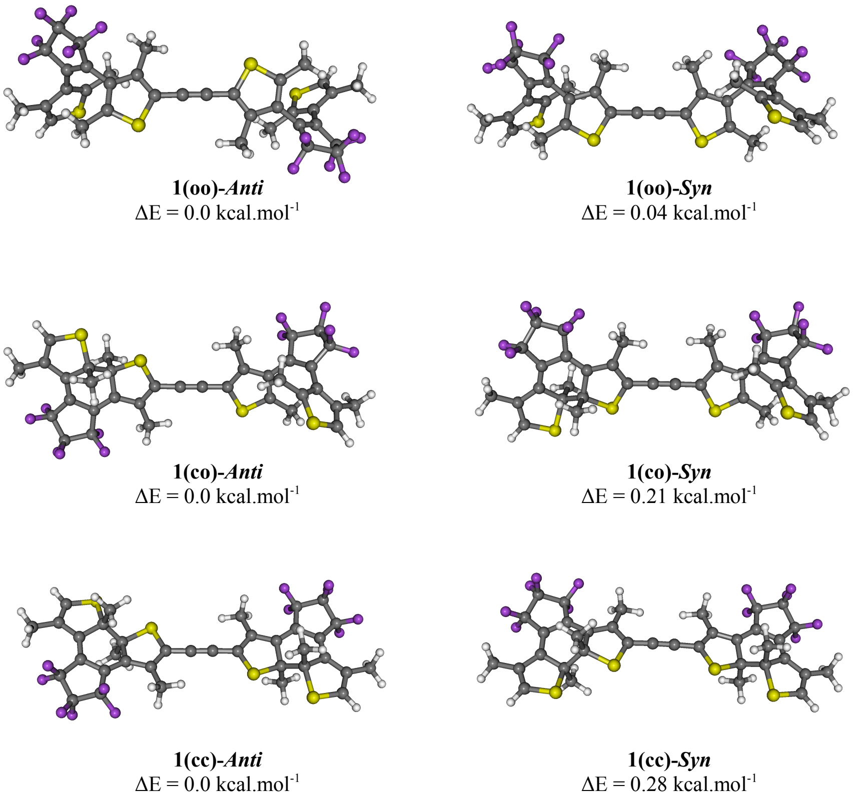

Figure S2: Stable conformers of **1**.

Basis set effect in the optical properties of dyad 1

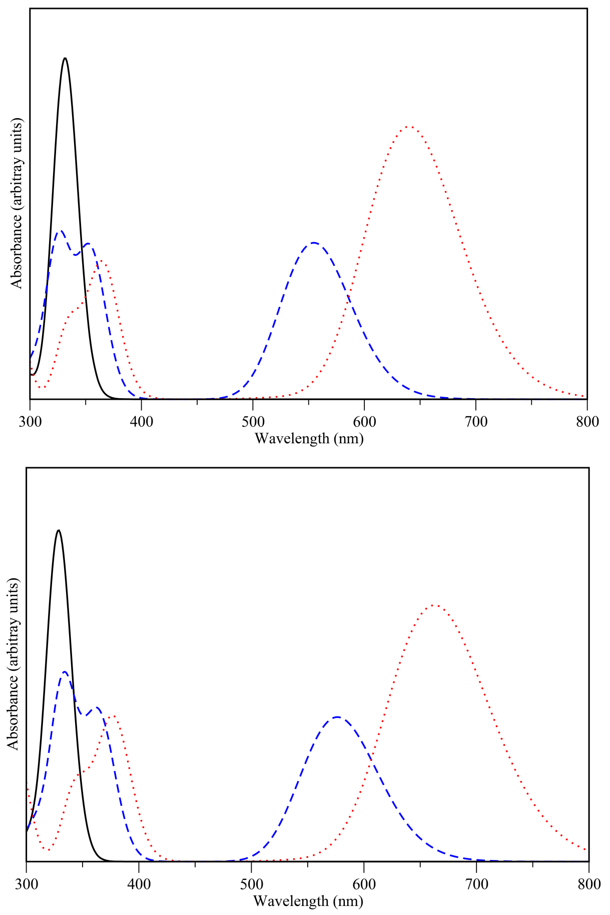

Figure S3: Theoretical spectra for compounds **1(oo)** (solid line), **1(co)** (dashed line) and **1(cc)** (dotted line), at the CAM-B3LYP/6-31G(d) (upper part) and CAM-B3LYP/6-311+G(2d,p) (lower part).

Table S2: Theoretical and experimental[3] optical properties for the different isomers of **1** in hexane. Wavelengths, oscillator strengths (f) and composition of the different transitions are given, along with the energy difference between experimental and theoretical results.

Isomer	method	λ (nm)	$\Delta E_{exp/theo}$ (eV)	f	composition
**1oo**	exp	320			
		ca. 270			
	CAM-B3LYP/	331	-0.12	1.00	HOMO→LUMO (88%)
	6-31G(d)	266	+0.07	0.08	HOMO-2→LUMO+1 (17%)
					HOMO-1→LUMO+2 (18%)
					HOMO→LUMO+6 (33%)
	CAM-B3LYP/	328	-0.09	1.00	HOMO→LUMO (63%)
		6-311+G(2d,p)			HOMO→LUMO+1 (16%)
					HOMO→LUMO+2 (14%)
		272	+0.03	0.09	HOMO-2→LUMO+1 (18%)
					HOMO-1→LUMO+2 (18%)
					HOMO→LUMO+3 (13%)
					HOMO→LUMO+6 (11%)
**1co**	exp	584			
		ca. 365			
	CAM-B3LYP/	555	+0.11	0.43	HOMO→LUMO (95%)
	6-31G(d)	355	+0.09	0.50	HOMO-2→LUMO (14%)
					H-1→LUMO (75%)
	CAM-B3LYP/	576	+0.03	0.49	HOMO→LUMO (94%)
	6-311+G(2d,p)	365	0.00	0.49	HOMO-2→LUMO (17%)
					HOMO-1→LUMO (71%)
**1cc**	exp	–	–	–	
	CAM-B3LYP/	640		0.93	HOMO→LUMO (86%)
	6-31G(d)				HOMO-1→LUMO+1 (13%)
		366		0.45	HOMO-2→LUMO (70%)
					HOMO-1→LUMO+1 (11%)
					HOMO-3→LUMO+1 (13%)
	CAM-B3LYP/	663		0.87	HOMO→LUMO (85%)
	6-311G+(2d,p)				HOMO-1→LUMO+1 (13%)
		377		0.48	HOMO-2→LUMO (68%)
					HOMO-1→LUMO+1 (11%)
					HOMO-3→LUMO+1 (14%)

Optical properties of a "normal-inverse" DTE dyad

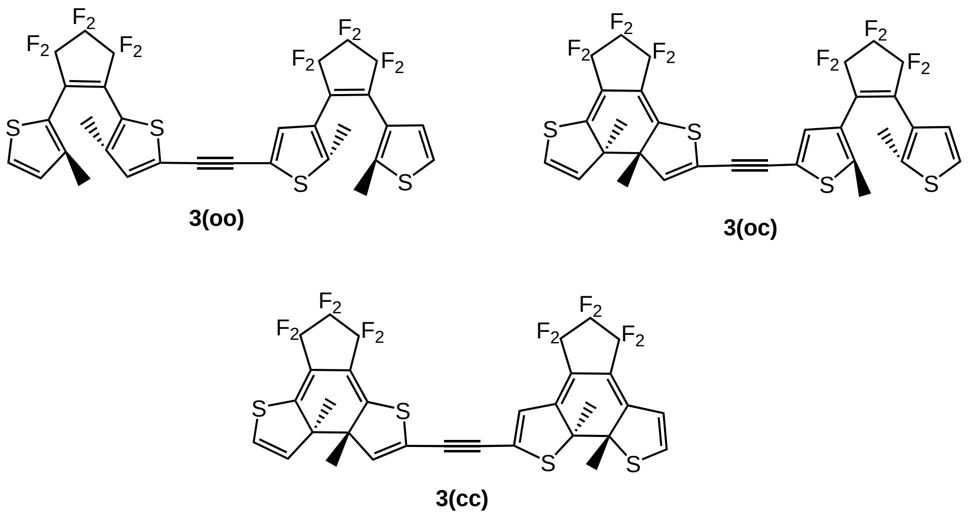

Scheme 1: Isomers of **3**.

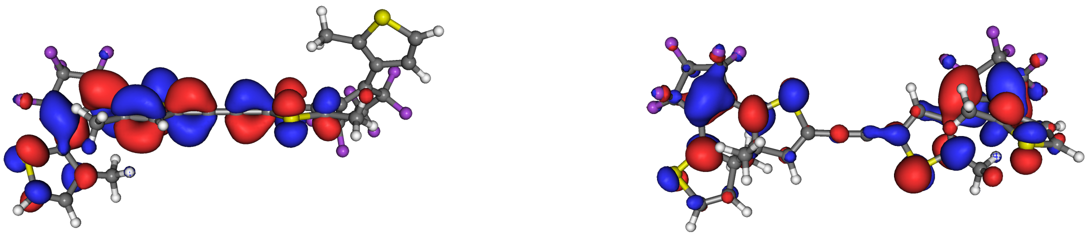

LUMO of **3(oo)** LUMO+1 of **3(co)**

Figure S4: Photochromic virtual molecular orbitals in **3(oo)** and **3(co)** (cut-off=0.02 a.u.).

The LUMO of **3(oo)** is centered on the inverse DTE and the HOMO→LUMO excitation should then selectively close this part of the dimer. In **3(co)**, the LUMO is the photochromic orbital centered on the open DTE, but none of the computed transition involving this orbital is considered as efficient (see the main text for the criteria).

Table S3: Optical properties of the different isomers of **3**, with a focus on the photochromic transitions.

isomer	photochromic orbital	photochromic state	λ (nm)	f	orbital composition
**3oo**	LUMO	S_1	362	1.00	HOMO→LUMO (90%)
**3(co)**	LUMO+1	S_2	309	0.83	HOMO→UMOL+1 (14%)
		S_4	281	0.23	HOMO-2→LUMO+1 (11%)
					HOMO-1→LUMO+1 (34%)
**3(cc)**		S_1	491	0.56	HOMO→LUMO (87%)

References

(1) Frisch, M. J. et al. Gaussian 09 Revision D.01. 2009; Gaussian Inc. Wallingford CT.

(2) TURBOMOLE V6.4 2014, a development of University of Karlsruhe and Forschungszentrum Karlsruhe GmbH, 1989-2007, TURBOMOLE GmbH, since 2007; available from `http://www.turbomole.com`.

(3) Kaieda, T.; Kobatake, S.; Miyasaka, H.; Murakami, M.; Iwai, N.; Nagata, Y.; Itaya, A.; Irie, M. Efficient Photocyclization of Dithienylethene Dimer, Trimer, and Tetramer: Quantum Yield and Reaction Dynamics. *J. Am. Chem. Soc* **2002**, *124*, 2015–2024.
